# Cognitive, Behavioral and Goal Adjustment Coping and Depressive Symptoms in Young People with Diabetes: A Search for Intervention Targets for Coping Skills Training

**DOI:** 10.1007/s10880-015-9417-8

**Published:** 2015-01-23

**Authors:** Vivian Kraaij, Nadia Garnefski

**Affiliations:** Department of Clinical Psychology, Leiden University, P.O. Box 9555, 2300 RB Leiden, The Netherlands

**Keywords:** Coping, Goal adjustment, Depression, Young people, Diabetes

## Abstract

The aim of the present study was to find relevant coping factors for the development of psychological intervention programs for young people with Type 1 (T1) diabetes. A wide range of coping techniques was studied, including cognitive coping, behavioral coping and goal adjustment coping. A total of 78 young people with T1 diabetes participated. They were contacted through a social networking website, several Internet sites, and flyers. A wide range of coping techniques appeared to be related to depressive symptoms. Especially the cognitive coping strategies self-blame, rumination, refocus positive, and other-blame, together with goal adjustment coping, were of importance. A large proportion of the variance of depressive symptoms could be explained (65 %). These findings suggest that these specific coping strategies should be part of coping skills trainings for young people with T1 diabetes.

## Introduction

Type 1 (T1) diabetes in youth is one of the most common chronic conditions of this age group (Compas, Jaser, Dunn, & Rodriguez, 2012; Pettitt et al., 2014; Stanescu, Lord, & Lipman, 2012). Recent research suggests that the incidence of T1 diabetes is increasing worldwide, with the peak age of onset at puberty (Compas et al., 2012; Pettitt et al., 2014; Stanescu et al., 2012). The disease puts extensive demands on young people, such as scheduling daily insulin injections, blood glucose monitoring, keeping a strict diet, regular physical exercise and management of hypoglycemia and hyperglycemia. These demands have an impact on all domains of life, including school and social life (Wysocki, Hough, Ward, & Green, 1992). Having T1 diabetes can therefore be considered as an important stressor in a developmental period where conformity is important and where youth struggle for independence (Boekaerts & Roder, 1999; Compas et al., 2012).

Studies show that young people with T1 diabetes are found to be at increased risk of developing emotional problems, such as depression (Berg et al., 2007; de Wit & Snoek, 2011). Furthermore, several studies on young people with T1 diabetes found consistent evidence that more depressive symptoms were associated with adverse health outcomes, such as less frequent blood glucose monitoring, poor glycemic control, and increased risk of hospitalization for diabetes-related reasons (Bernstein, Stockwell, Gallagher, Rosenthal, & Soren, 2013; Johnson, Eiser, Young, Brierley, & Heller, 2013). It can be concluded that it is essential that improvement of well-being becomes a major treatment goal for young people with T1 diabetes.

A wide range of behaviorally oriented interventions have been developed that focus on diabetes treatment adherence, such as Behavioral Family Systems Therapy and Family-Focused Teamwork Intervention (for a review see Anderson, Svoren & Laffel, 2007; Delamater et al., 2001; Wysocki, 2006). Review studies show that various interventions were effective in improving several dimensions of functioning, but the overall impact of these interventions on glycemic control was moderate (Anderson et al., 2007; Wysocki, 2006). Furthermore, several intervention programs for youngsters have been developed to manage stress related to T1 diabetes. These programs consisted of coping skills training. In these programs emphasis was placed on developing the coping skills of social problem solving, social skills training, cognitive behavior modification, and conflict resolution (Grey, Boland, Davidson, Yu, & Tamborlane, 1999). In general, youngsters in these intervention programs improved on coping and quality of life (Ambrosino et al., 2008; Grey, Boland, Davidson, Li, & Tamborlane, 2000; Grey et al., 2009; Whittemore et al., 2012). However, when comparing the effect of these interventions to control groups following diabetes education or diabetes management, findings were less strong and not significant as both intervention and control group improved on quality of life over time (Ambrosino et al., 2008; Grey et al., 2009; Whittemore et al., 2012). To maximize the effectiveness of coping skills interventions, intervention targets should be included that are proven to be of importance in relation to quality of life. Therefore, studies should be conducted that focus on the specific coping skills that are related to well-being. The current study focuses on the relationship between a wide range of coping strategies and depressive symptoms in young people with Type 1 diabetes in order to find relevant, evidence-based, intervention targets.

Several studies have been performed on the way young people cope with T1 diabetes. Coping strategies such as primary control coping and secondary control coping (Jaser & White, 2010), cognitive restructuring and social support (Edgar & Skinner, 2003) have been found to be related to a better quality of life in these young people. Coping strategies such as self-criticism, blaming others, wishful thinking (Grylli, Wagner, Hafferl-Gattermayer, Schober, & Karwautz, 2005), mental disengagement, behavioral disengagement, aggressive coping and self-blame (Graue, Wentzel-Larsen, Bru, Hanestad, & Sovik, 2004) have been found to be related to poorer quality of life in this group. A few studies focused on specific cognitive and behavioral coping strategies in young people with a chronic disease. These studies found that especially self-blame (Kraaij & Garnefski, 2012), rumination and catastrophizing (Garnefski, Koopman, Kraaij, & ten Cate, 2009; Kraaij & Garnefski, 2012) were the most important “predictors” of psychological maladjustment in young people with a chronic disease. Other cognitive and behavioral coping strategies, such as positive refocusing, putting into perspective, blaming others, active coping, and asking for emotional support, also correlated significantly with depressive symptoms in young people with a chronic medical condition (Kraaij & Garnefski, 2012).Most of these coping strategies have not been studied in a large sample of youth with diabetes. In the present study we will focus on a broad range of specific cognitive and behavioral coping strategies and their relationship with depressive symptoms, as they fit well within the well-established cognitive behavior therapies.

Another way to cope with having a chronic medical condition such as T1 diabetes is through goal adjustment. Goals give people a sense of identity and meaning in life and are therefore believed to be of great importance for psychological well-being (Austin & Vancouver, 1996; Emmons, 2003). Several studies have shown that a chronic medical condition may strongly interfere with the attainment of goals (Boersma, Maes, & Joekes, 2005; Van der Veek, Kraaij, Van Koppen, Garnefski, & Joekes, 2007). When people are confronted with obstacles in the attainment of their goals, this may result in reduced well-being (Heckhausen, Wrosch, & Fleeson, 2001; Kraaij et al., 2008; Kraaij, Garnefski, & Schroevers, 2009; Massey, Gebhardt, & Garnefski, 2008; Tunali & Power, 2002; Wrosch & Scheier, 2003; Wrosch, Scheier, Miller, Schulz, & Carver, 2003). One way to restore well-being is to withdraw effort and commitment from goals that are no longer attainable, and to reengage in alternative meaningful goals. To our knowledge, no studies have been performed on the relationship between goal adjustment coping and well-being in young people with T1 diabetes. One study focusing on young people with a variety of chronic medical conditions, showed that those who were better in goal adjustment coping, displayed fewer depressive symptoms (Kraaij & Garnefski, 2012). In the present study we will examine whether goal adjustment coping in young people with T1 diabetes is related to symptoms of depression.

More research is needed to find relevant coping techniques for the development of evidence-based psychological treatment programs for young people with T1 diabetes. Specifically, we will examine the relationship between cognitive coping, behavioral coping, goal adjustment coping and symptoms of depression in young people with T1 diabetes. First, we will examine the bivariate relationships between coping and depressive symptoms. Next, the multivariate relationships of the coping techniques with depressive symptoms will be studied. Based on the literature, we hypothesize that self-blame, rumination and catastrophizing will be the most important “predictors” of depressive symptoms. More specifically we hypothesize that an increase in use of these strategies will be associated with higher depression scores. In addition, we hypothesize that also a higher score on blaming others will be associated with a higher depression score. In contrast, we hypothesize that a higher use of positive refocusing, putting into perspective, active coping, and asking for emotional support, will be associated with lower depression scores. Finally, we hypothesize that an increase in use of goal adjustment coping will be associated with lower depression scores.

## Methods

### Sample

The sample comprised of 78 young people with T1 diabetes. Thirty-five percent were male, and the mean age was 19.49 (SD 2.3; range 16–23). The majority (52.5 %) of the young people attended college or university; the others attended higher general secondary education or pre-university education (20.5 %), intermediate vocational education (9.0 %), or lower secondary education (6.4 %). The remaining young people were either working (10.3 %) or staying in an institution (1.3 %). Half of the sample (55.1 %) was living at home, and the other half (44.9 %) was living independently.

It is noteworthy that in two respects, the sample’s demographics differed from those of the general population. First, more females (*n* = 51) than males (*n* = 27) participated. Even though females have been found to have slightly higher prevalence rates of diabetes (2.30 case subjects/1,000) than males (2.16 case subjects/1,000; Pettitt et al., 2014), males were markedly underrepresented in the present study. In addition, highly educated persons are overrepresented in the sample. When compared to national statistics (Centraal Bureau voor de Statistiek, 2014), the present sample contains a greater proportion of highly educated young people than in the population at large; 53 % of participants attended college or university as compared to 33 % nationally.

Respondents reported they had T1 diabetes on average since they were 12 years old (*M* = 11.85; SD 4.8; range 2–22 years of age). They suffered from T1 diabetes on average for 8 years (*M* = 7.63; SD 4.7; range 0–17.5 years). Half (48.7 %) of the sample reported they used insulin injections, the other half (51.3 %) used an insulin pump. Nineteen percent of the respondents reported they had stable blood sugar levels in general, 63 % reported they had periods of stable and unstable blood sugar levels and the remaining 18 % reported they generally had unstable levels. Respondents reported they experienced (occasional or permanent) goal frustration (not being able to do something desirable because of having diabetes) at various domains: at school or work (49 %); at home (32 %); with peers/friends (60 %); with their hobbies (41 %); and with sport activities (60 %).

### Procedure

After obtaining permission from the Ethics Committee of the University, participants were recruited through various pathways. First, a Dutch social networking website was used, which has more than 9 million registered users and is very popular among young people. Furthermore, several Internet sites related to T1 diabetes patients were used to contact young people with T1 diabetes. In addition, flyers were distributed and posted at several locations including: the University, several dormitories, high schools, hospitals, and flyers were also given to diabetes nurses in the vicinity, in order to reach as many young people with T1 diabetes as possible. All young people with T1 diabetes between the ages of 16 and 23 years could participate. The call for participation contained the Internet address where young people could find information about the study and a link to the online questionnaire. On the website, people were told that if they participated, that they could win one of three rewards, either a digital camera, an iPod shuffle, or a gift certificate of €20. All respondents were informed about the study at the beginning of the questionnaire. They were told that they were free to decide whether they wanted to participate and that they could stop at any time without giving a reason. They were also told that anonymity was guaranteed and that their email address would only be used for the raffle for the three rewards. After this information, respondents could tick a box next to the following statement “I read and understood the information provided above, and give permission to use my data anonymously.” After ticking this box, respondents got access to the following questions.

### Measures

#### Cognitive Coping

To measure cognitive coping strategies the Cognitive Emotion Regulation Questionnaire was used (CERQ; Garnefski, Kraaij, & Spinhoven, 2001; Garnefski, Kraaij, & Spinhoven, 2002). The CERQ consists of 36 items and 9 subscales, and assesses what people think at the time of or after the experience of threatening or stressful life events. In the present study, respondents were asked which specific cognitive coping strategies they used in relation to having T1 diabetes, by the instruction: “You have diabetes. There are more people who experience this and everyone deals with this in his or her own way. By means of the following questions, you are asked what you think about having diabetes. Please read the sentences below and indicate how often you have the following thoughts.” Each subscale consists of four items. Each of the items has a five-point Likert scale, with values that range from 1 = (almost) never to 5 = (almost) always. A subscale score can be obtained by adding up the four items, indicating the extent to which a certain cognitive coping strategy is used (possible scores for each subscale ranged from 4 to 20). The CERQ subscales are: self-blame, which refers to thoughts of blaming yourself for your diabetes (e.g., “I think to myself that I am to blame for everything,” and “I think that I am the one who is responsible for my diabetes”); acceptance, which refers to thoughts of accepting your diabetes and resigning yourself to it (e.g., “I think that I have to accept my diabetes,” and “I think that I must learn to live with diabetes”); rumination, which refers to thinking about the feelings and thoughts associated with the diabetes (e.g., “I am preoccupied with what I think and feel about my diabetes,” and “I often think about how I feel about having diabetes”); positive refocusing, which refers to thinking about joyful and pleasant issues instead of thinking about the diabetes (e.g., “I think of something nice instead of my diabetes,” and “I think about pleasant experiences”); refocus on planning, which refers to thinking about what steps to take and how to handle the diabetes (e.g., “I think of what I can do best,” and “I think about how to change the situation”); positive reappraisal, which refers to thoughts of attaching a positive meaning to the diabetes in terms of personal growth (e.g., “I think I can learn something from my diabetes,” and “I think that I can become a stronger person as a result of my diabetes”); putting into perspective, which refers to thoughts of playing down the seriousness of the diabetes or comparing it relative to other events that are worse (e.g., “I think that it all could have been much worse,” and “I think that other people go through much worse experiences”); catastrophizing, which refers to thoughts of explicitly emphasizing the terror of the diabetes (e.g., “I continually think how horrible my diabetes is,” and “I often think that having diabetes is the worst that can happen to a person”); and other-blame, which refers to thoughts of putting the blame for the diabetes on others (e.g., “I think about the mistakes others have made in this matter,” and “I feel that others are to blame for it”). The psychometric properties of the CERQ were proven to be good (Garnefski & Kraaij, 2006; Garnefski, Baan, & Kraaij, 2005; Garnefski et al., 2001; Garnefski et al., 2002; Kraaij & Garnefski, 2006; Kraaij, Garnefski, & Van Gerwen, 2003), with Cronbach’s alpha coefficients in most cases well over .70 and in many cases even over .80. Furthermore, the CERQ has been shown to have good factorial validity, good discriminative properties and good construct validity (Garnefski et al., 2002). In the present study the alpha-reliabilities of the subscales also appeared to be good, with alphas ranging from .74 to 87 (see Table 1).

**Table 1:** Reliabilities, means, standard deviations (SD) and Pearson correlation matrix for study variables

	Cron-bach’s alpha	Mean (*SD*)	1	2	3	4	5	6	7	8	9	10	11	12
1. Depression	.95	31.6 (14.2)	–											
2. Self-blame	.74	7.8 (3.2)	.65***	–										
3. Acceptance	.86	11.9 (4.5)	.26*	.28*	–									
4. Rumination	.83	8.5 (3.5)	.49***	.34**	.57***	–								
5. Refocus positive	.87	13.7 (4.2)	−.48***	−.29*	.16	−.16	–							
6. Refocus planning	.82	11.6 (3.8)	−.01	.02	.51***	.42***	.32**	–						
7. Positive reappraisal	.86	10.2 (4.6)	−.24*	−.20	.33**	.25*	.38**	.61***	–					
8. Putting into perspec	.87	12.3 (4.3)	−.22	−.02	.42***	−.04	.49***	.47***	.54***	–				
9. Catastrophizing	.79	6.0 (2.5)	.29*	−.07	.17	.47***	−.25*	.04	.04	−.24*	–			
10. Other-blame	.75	4.5 (1.6)	.26*	.15	.16	.09	.10	.04	−.02	−.05	.11	–		
11. Active coping	.73	9.1 (2.8)	−.08	−.13	.42***	.38**	.21	.63***	.51***	.30**	.14	−.11	–	
12. Emotional support	.84	7.8 (2.9)	−.04	−.03	.43***	.30*	.36**	.35**	.39**	.23	.26*	.27*	.48***	–
13. Goal adjustment	.87	12.1 (4.5)	−.40***	−.11	.07	−.18	.38**	.15	.19	.36**	−.32**	.02	.07	.06

* *p* < .05; ** *p* < .01; *** *p* < .001

#### Behavioral Coping

To measure behavioral coping strategies two subscales of the COPE were used (Carver, Scheier, & Weintraub, 1989), reflecting pure behavioral strategies: active coping and use of emotional social support. In the scale use of emotional social support, two items were slightly rephrased to emphasize the behavior: “I try to get emotional support from friends or relatives” was changed into “I ask for emotional support from friends or relatives,” and “I get sympathy and understanding from someone” was changed into “I look for sympathy and understanding from someone.” Example items of active coping are: “I do what has to be done, one step at a time,” and “I take direct action to get around the problem.” Each subscale consists of four items. Each of the items has a four-point Likert scale, with values that range from 1 = rarely or never to 4 = very often. A subscale score can be obtained by adding up the four items, indicating the extent to which a certain behavioral coping strategy is used (possible scores for each subscale range from 4 to 16). Good psychometric properties have been found in the past (Carver et al., 1989). In the present study the alpha-reliabilities of the subscales also appeared to be good, with an alpha of .73 for active coping and .84 for use of emotional social support.

#### Goal Adjustment Coping

Goal disengagement and reengagement were measured by the Adolescent Goal Obstruction Questionnaire (A-GOQ; Garnefski & Kraaij, n.d.). The A-GOQ measures what young people do when important things (goals) they want to do (on various domains) are not possible due to a stressor (goal frustration), in this case having T1 diabetes. The goal disengagement subscale measures the extent to which one considers oneself able to withdraw effort and commitment from these unattainable goals (e.g., “I can accept it,” and “I can get over it”), while the goal reengagement subscale measures the extent to which one considers oneself able to reengage in alternative meaningful goals, in case that preexisting goals can no longer be reached (e.g., “I look for something else that I can do,” and “I have confidence that I find other things that I can do”). Both subscales consist of two items. Each of the items has a five-point Likert scale that ranged from 1 = (almost) never to 5 = (almost) always. A subscale score can be obtained by adding up the items. In the present study the two subscales were highly correlated (*r* = .61, *p* < .001) and were therefore combined into the scale goal adjustment, by summing all four items (possible scores ranged from 4 to 20). A high score reflects better goal adjustment. Cronbach’s alpha of this scale was .87.

#### Depressive Symptoms

Depressive symptoms were measured by the depression subscale of the Symptom Check List (SCL-90; Derogatis, 1977; Dutch translation and adaptation by Arrindell & Ettema, 1986). This subscale consists of 16 items, assessing whether and to what extent the participants report symptoms of depression (e.g., “feeling low in energy,” “feeling hopeless about the future,” and “feelings of worthlessness”). Answer categories of the items range from 1 = not at all to 5 = very much. The scale scores are obtained by adding up the items belonging to the scale items (possible scores ranged from 16 to 80). A high score reflects more depressive symptoms. Psychometric properties of the SCL-90 have been found to be good, with Cronbach’s alpha coefficients for depression ranging from .82 to .93. Furthermore, the SCL-90 has been shown to have good factorial validity and good construct validity (Arrindell & Ettema, 1986; Derogatis & Cleary, 1977). In the present sample the Cronbach’s alpha was .95.

### Statistical Analyses

In our analyses we used the raw scores of the coping scales and depression scale. To study the relationships between cognitive coping, behavioral coping, goal adjustment coping and depressive symptoms, Pearson correlations and multiple regression analysis (MRA) were used. In the MRA the “simultaneous method” was used where all specified variables were included in the regression equation (called “method enter” in SPSS). Because the sample size was not large enough to include all variables in the MRA, only the coping strategies which had a significant correlation with depressive symptoms were included in the MRA.

## Results

### Preliminary Analyses

Prior to performing the main analyses, means, standard deviations and alpha reliabilities of the study variables were calculated and are shown in the first two columns of Table 1. Furthermore, Pearson correlations among the study variables were computed (Table 1). The magnitude of correlations among the measures of cognitive coping, behavioral coping, and goal adjustment coping were not so high as to indicate that multicollinearity might be problematic for the MRA (see Tabachnick & Fidell, 1996). Age, gender, living situation (at home or independently) and using insulin injections versus an insulin pump were not significantly related to depression. Therefore, there was no need to control for these variables in the main analyses.

### Relationships Between Coping and Depressive Symptoms

To study the relationships between coping and depressive symptoms, Pearson correlation coefficients were calculated (Table 1). Various cognitive coping strategies were significantly related to depressive symptoms. Self-blame, acceptance, rumination, catastrophizing, and other-blame were positively correlated with depressive symptoms, while refocus positive and positive reappraisal were negatively correlated with depressive symptoms. None of the behavioral coping strategies (active coping and emotional support) had a significant correlation with depressive symptoms. Finally, goal adjustment had a significant negative relationship with depressive symptoms.

Next, a multiple regression analysis was performed (Table 2). Coping strategies which had a significant correlation with depressive symptoms were included. Self-blame, rumination and other-blame had a positive significant relationship with depressive symptoms, whereas refocus positive and goal adjustment had a negative significant relationship with depressive symptoms. Sixty-five percent of the variance was explained.

**Table 2:** Multiple regression analysis of coping strategies on depressive symptoms (method enter)

	B	SE B	β
Self-blame	1.95	.40	.43***
Acceptance	.23	.31	.07
Rumination	.89	.44	.22*
Refocus positive	−.69	.30	−.20*
Positive reappraisal	−.37	.27	−.12
Catastrophizing	.31	.57	.05
Other-blame	1.61	.66	.18*
Goal adjustment	−.68	.26	−.21*
R^2^/adjusted R^2^			.69/.65
F (df)			17.17*** (8.63)

* *p* < .05; ** *p* < .01; *** *p* < .001

## Discussion

Although young people with T1 diabetes report more depressive symptoms and may benefit from intervention, only a limited number of studies have focused on which coping factors could be useful in shaping coping skills interventions. The present study included a wide range of coping techniques. The aim was to study the relationships between cognitive coping, behavioral coping, goal adjustment coping, and symptoms of depression in young people with T1 diabetes, in order to find relevant, evidence-based, intervention targets.

First, the bivariate relationships were studied. An interesting finding is that a wide range of coping techniques appeared to be related to depressive symptoms. We already knew from earlier research that several coping strategies were related to psychological problems in young people with diabetes (Edgar & Skinner, 2003; Graue et al., 2004; Grylli et al., 2005; Jaser & White, 2010). The present study adds to this knowledge that even a wider range of coping strategies seems to be important in relation to the level of depression in young people with T1 diabetes. These coping strategies could be useful evidence-based intervention targets. When looking closer at the different coping strategies, it is interesting to see that the cognitive coping strategies were especially important: self-blame, acceptance, rumination, positive refocusing, positive reappraisal, catastrophizing, and blaming others were all related to the level of depressive symptoms. In contrast, none of the behavioral coping strategies were significantly related to depression. Both active coping and asking for emotional support were included in the present study since they were significantly related to depressive symptoms in an earlier study among young people with various medical conditions (Kraaij & Garnefski, 2012). This finding however, could not be confirmed by the present study. Overall, these results partially confirm our hypotheses. The findings suggest that cognitive coping strategies have a stronger influence than behavioral coping strategies on symptoms of depression in young people with T1 diabetes. Possibly this has to do with the fact that Type 1 diabetes is a chronic disease, and no action can be taken that will cure it. Coping in cognitive ways may be more effective methods for successfully adjusting to living with a chronic condition. It would be interesting to see if behavioral coping strategies are more important for young people with an illness that can be cured and for which they can actually do something to change their condition. If the findings of the present study can be confirmed, they would have important clinical implications, namely, they suggest the possibility that interventions for young people with diabetes should focus primarily on cognitive coping strategies. Goal adjustment coping also had a significant relationship with depressive symptoms, thereby confirming our hypothesis. This finding suggests that the emotional well-being of young people with T1 diabetes could be facilitated by interventions that strengthen their ability to disengage from goals that are no longer obtainable, and instead pursue alternative goals that are attainable. This finding on goal adjustment is in line with studies focusing on adult samples (Wrosch & Scheier, 2003; Wrosch et al., 2003), and one study on young people with various chronic medical conditions (Kraaij & Garnefski, 2012). Respondents in the present study reported goal frustration on all domains, with greatest frustration in activities involving peers and friends and also sports activities. Further studies are needed that examine the process of goal frustration and subsequent goal adjustment.

The regression analysis focused on multivariate relationships and showed that self-blame, rumination, positive refocusing, blaming others and goal adjustment were significantly associated with level of depression. Contrary to expectation, catastrophizing was not a significant factor in the multivariate prediction equation. Taken as a whole, the results show that, among young people with T1 diabetes, individuals who report fewer symptoms of depression also have: fewer thoughts of blaming themselves, or blaming others for having diabetes; fewer ruminative thoughts about their diabetes; have more thoughts about other, more pleasant matters; and do better in adjusting their life goals. With these coping techniques, a large proportion of the variance of depressive symptoms could be explained (65 %). The findings of the present study suggest that these specific coping strategies are relevant for the development of evidence-based psychological treatment programs specifically tailored for young people with T1 diabetes. Although various successful interventions have been developed for young people with diabetes, such as Behavioral Family Systems Therapy and Family-Focused Teamwork Intervention (Anderson, 2007; Delamater et al., 2001; Wysocki, 2006) and coping skills training (Ambrosino et al., 2008; Grey et al., 2000; Grey et al., 2009; Whittemore et al., 2012), the findings of the present study might be of additional value. Coping skills training could be adapted or developed that include and highlight intervention components that focus on the cognitive coping strategies and goal adjustment coping found in the present study. Adding such components to training programs might further increase those programs effectiveness to enhance the ability of young people with T1 diabetes to cope in optimal ways with their chronic condition, and thereby reduce depressive symptoms. Future studies should look at the effectiveness of intervention programs for young people with T1 diabetes that incorporate such evidence-based intervention. A similar approach has been used with adults with various medical conditions. Self-help programs were developed after the completion of a series of studies focusing on a wide range of coping strategies and subsequent studies showed that these programs were indeed effective in reducing symptoms of depression (Garnefski, Kraaij, Benoist, Bout, Karels & Smit, 2013; Garnefski, Kraaij, & Schroevers, 2011; Kraaij et al., 2010). Following a similar strategy in the development of an evidence-based intervention for youth with diabetes could be a promising approach.

The present study has methodological shortcomings that further studies must address. The first issue of concern is the small sample size and the representativeness of the group studied. The sample was obtained through several Internet sites and through flyers which were spread at various places. As noted elsewhere, the participants contained a disproportionate share of females and of highly educated individuals. The second limitation of the present study results from the fact that coping and depressive symptoms were measured at the same point in time. Therefore, no conclusions can be drawn regarding the causality or temporal order of these variables. In order to solve these cause and effect issues, these aspects should be studied longitudinally. Another limitation of the design was that all variables were measured by self-report instruments, which may have caused some bias. No objective diabetes information, such as HbA1c values and objective information about glycemic control, was available, making it difficult to characterize the sample and to study the actual impact on objective health outcomes. Furthermore, goal adjustment was measured with four items only. Since goal adjustment seemed to be a relevant coping strategy for young people with a chronic disease, it would be important to develop a more comprehensive measure of this concept for future studies. Finally, several aspects that could also be related to symptoms of depression, such as socio economic status, social support and personality characteristics were not included in the present study. Future studies should try to include these other issues as well.

Despite these shortcomings, a wide variety of coping strategies seem to be related to symptoms of depression in young people with T1 diabetes. Especially the cognitive coping strategies self-blame, rumination, refocus positive, and other-blame seem to be important factors, together with goal adjustment coping. If these findings can be confirmed, they could contribute to the focus and content of coping skills programs for young people with T1 diabetes.
